# Epigenetic Inactivation of Acetyl-CoA Acetyltransferase 1 Promotes the Proliferation and Metastasis in Nasopharyngeal Carcinoma by Blocking Ketogenesis

**DOI:** 10.3389/fonc.2021.667673

**Published:** 2021-08-16

**Authors:** Yunliang Lu, Xiaohui Zhou, Weilin Zhao, Zhipeng Liao, Bo Li, Peipei Han, Yanping Yang, Xuemin Zhong, Yingxi Mo, Ping Li, Guangwu Huang, Xue Xiao, Zhe Zhang, Xiaoying Zhou

**Affiliations:** ^1^Department of Otolaryngology-Head & Neck Surgery, First Affiliated Hospital of Guangxi Medical University, Nanning, China; ^2^Life Science Institute, Guangxi Medical University, Nanning, China; ^3^Department of Radiotherapy, First Affiliated Hospital of Guangxi Medical University, Nanning, China; ^4^Department of Research, Affiliated Tumor Hospital of Guangxi Medical University, Nanning, China; ^5^Department of Pathology, Affiliated Stomatological Hospital of Guangxi Medical University, Nanning, China

**Keywords:** nasopharyngeal carcinoma, ACAT1, proliferation, metastasis, ketogenesis

## Abstract

The dysregulation of epigenetic modification and energy metabolism cooperatively contribute to the tumorigenesis of nasopharyngeal carcinoma (NPC). However, the detailed mechanisms underlying their joint contribution to NPC development and progression remain unclear. Here, we investigate the role of Acy1 Coenzyme A Acyltransferases1 (ACAT1), a key enzyme in the metabolic pathway of ketone bodies, in the proliferation and metastasis of NPC and to elucidate the underlying molecular mechanisms. Ketogenesis, plays a critical role in tumorigenesis. Previously, we reported two enzymes involved in ketone body metabolism mediate epigenetic silencing and act as tumor suppressor genes in NPC. Here, we identify another key enzyme, Acetyl-CoA acetyltransferase 1 (ACAT1), and show that its transcriptional inactivation in NPC is due to promoter hypermethylation. Ectopic overexpression of ACAT1 significantly suppressed the proliferation and colony formation of NPC cells *in vitro*. The migratory and invasive capacity of NPC cells was inhibited by ACAT1. The tumorigenesis of NPC cells overexpressing ACAT1 was decreased *in vivo*. Elevated ACAT1 in NPC cells was accompanied by an elevated expression of CDH1 and a reduced expression of vimentin and SPARC, strongly indicating that ACAT1 is involved in regulating epithelial-mesenchymal transition (EMT). We also found that ACAT1 contributes to increased intracellular levels of β-hydroxybutyrate (β-HB). Exogenously supplied β-HB significantly inhibits the growth of NPC cells in a dose-dependent manner. In summary, ACAT1 may function as a tumor suppressor *via* modulation of ketogenesis and could thus serve as a potential therapeutic target in NPC. In summary, our data suggest that regulation of ketogenesis may serve as adjuvant therapy in NPC.

## Introduction

Aberrant energy metabolism was defined as one of the hallmarks of cancer (1). The metabolic reprogramming in cancer includes the generation of additional energy, providing more substrates for biosynthesis, and rebalancing cellular redox status (2), thus facilitating the adaption of tumor cells to the tumor microenvironment and generating extra energy to sustain their malignant growth (3–6).

Nasopharyngeal carcinoma (NPC) derives from the epithelium of the nasopharynx. It is the most common one of the head and neck cancers prevalent in southern China and Southeast Asia (7). The synergistic effects of genetic susceptibility, Epstein-Barr virus (EBV) infection, and environmental carcinogens are considered to be the main etiologic factors of NPC (8). More than 75% of NPC patients were at an advanced stage when diagnosed in the clinic (9). The intensity-modulated radiotherapy (IMRT) has improved the disease control of NPC but local recurrence and distant metastasis remain therapeutic challenges (10). Therefore, further insights into the carcinogenesis of NPC are necessary to develop new therapeutic approaches.

In a previous study, we found that lipid droplets (LDs) accumulate in NPC, and the amount of intracellular LDs is associated with the growth and motility of NPC (11), indicating that an aberrant lipid metabolism contributes to the malignant behavior of tumor cells. Lipid synthesis is driven by the elongation of fatty acid chains with an activated acetyl group, in the form of Acetyl-CoA. Ketone body metabolism, a central node in physiological homeostasis, is one of the processes by which Acetyl-CoA is produced. Recently, we reported that a ketone body biosynthesis enzyme, 3-hydroxybutyrate dehydrogenase type 2 (BDH2), was significantly inactivated in NPC (12). In addition, hydroxymethylglutaryl-CoA lyase (HMGCL), an essential rate-limiting enzyme in ketogenesis, was found to be downregulated in NPC. Overexpression of HMGCL restores the production of β-hydroxybutyrate (β-HB), the main component of ketone bodies, thereby impeding NPC cell proliferation and metastasis (13). Our data highlights a remarkably reprogrammed ketone body metabolism in NPC, which warrants further investigation. The enzymes responsible for this might be considered as novel candidate targets for NPC therapy.

Acetyl-CoA acetyltransferase 1 (ACAT1) is located in mitochondria and catalyzes the reversible formation of acetoacetyl-CoA from two molecules of acetyl-CoA, as the first step of ketogenesis. To date, most studies have focused on mutations of ACAT1 leading to β-ketothiolase deficiency (14). Publications about the dysregulation of ACAT1 and its role in tumor pathogenesis are rare. The expression of ACAT1 was found to be decreased in clear cell renal cell carcinoma (ccRCC) (15). Restoring its expression significantly reduced the malignant behavior of ccRCC cells *in vitro* (16), suggesting that ACAT1 might be a tumor suppressor. On the contrary, ACAT1 was elevated in aggressive prostate cancer and was used as an effective diagnostic and prognostic biomarker (17, 18). The role of ACAT1 in NPC remains unclear.

In the present study, we investigated the abnormal expression of ACAT1 in NPC, revealing a role for ACAT1 in the biological behavior of this tumor, and related to the underlying molecular mechanisms associated with altered ketone body metabolism.

## Materials and Methods

### Cell Lines and Tissue Samples

NPC cell lines (C666-1, HONE1, CNE1, HK1, 5-8F, 6-10B, and TW03) were maintained in DMEM media (Invitrogen, USA) containing 10% fetal bovine serum (Invitrogen, USA), 100 U/mL penicillin, and 100 μg/mL streptomycin in a humidified incubator with 5% CO_2_ at 37°C.

In all, primary NPC tumor tissues were obtained from 42 diagnosed and untreated cases, all with informed consent from the donors, in the Department of Otolaryngology-Head and Neck Surgery, First Affiliated Hospital of Guangxi Medical University (Nanning, China). The diagnoses were established by experienced pathologists according to the World Health Organization (WHO) classification. We also included 36 normal nasopharyngeal epithelial tissues as control.

### Antibodies, Plasmids, and Transfection

Antibody to proteins of interest, source and dilutions used were as follows: ACAT1 (1:1000, HPA004428, Sigma, USA), β-catenin (1:1000, sc-376841, Santa Cruz, USA) and E-cadherin (1:1000, #3195P), Vimentin (1:1000, #5741P) and GAPDH (1:10000 #5174P) were purchased from Cell Signaling Technology.

The demethylating reagent 5-aza-dC was purchased from Sigma (#A3656, USA). Full-length ACAT1 cDNA (Origene, USA) was subcloned into the pCMV6-Entry vector (Origene, USA). 2μg pCMV6-Entry or pCMV6-ACAT1 plasmids were used in a 48 hrs transfection protocol using an overnight culture of NPC cells at 70–90% confluence in 6-well dishes, using an X-treme GENE HP DNA Transfection Reagent (Roche, Germany).

### Real-Time RT-PCR

Briefly, first-strand complementary DNA was synthesized using a First-Stand Reverse Transcription System (Transgene, Beijing, China). Real-time RT-PCR was carried out using SYBR Green PCR master mix in Step One Plus System (Applied Biosystems, USA). The primer sequences and cycling conditions for all experiments were as follows, ACAT1-F 5’-GGCTGGTGCAGGAAATAAGA-3’, ACAT1-R 5’ GGAATCCCTGCCTTTTCAAT-3’; GAPDH-F 5’-GCTCAGACACCATG-GGGAAG-3’, GAPDH-R 5’-TGTAGTTGAGGTCAATGAAGGGG-3’. GAPDH was used as an internal control. The PCR reaction conditions were as follows: 95°C for 10 min, followed by 40 cycles of 95°C for 15 s, and 60°C for 60 s. The relative gene expression was calculated using the comparative threshold cycle (2^-△△CT^) equation. All the experiments were performed in triplicate.

### Immunohistochemical Staining

After deparaffinization and rehydration, the antigen was retrieved in 5% urea buffer by microwave heating for 5 min, and then incubated in 3% H_2_O_2_ for 30 min to block endogenous peroxidase activity. Tissue sections were incubated with the primary antibody overnight at 4°C and followed by a secondary antibody for 1 h at room temperature. Subsequently, 3,3’-diaminobenzidine (DAB) reagent (ZLI-9018, ZSGB-BIO, Beijing) was used for the peroxidase reaction and hematoxylin was used for counterstaining. Images were acquired with a microscope (Olympus C-5050, Japan).

### Bisulfite Sequencing

The DNA methylation level was analyzed by MethylTarget^®^ (Genesky Biotechnologies Inc., Shanghai, China), a Next Generation Sequencing-based multiple Targeted CpG methylation analysis method. Genomic DNA (400ng) was subjected to sodium bisulfite treatment using EZ DNA Methylation™-GOLD Kit (Zymo Research) according to the manufacturer’s protocols. Multiple-PCR was performed with optimized primer set combinations. A 20 µl PCR reaction mixture was prepared for each reaction and included 1× reaction buffer (Takara), 3 mM Mg^2+^, 0.2 mM dNTP, 0.1 µM of each primer, 1U HotStarTaq polymerase (Takara) and 2 µl template DNA. The cycling program was 95°C for 2 min; 11 cycles of 94°C for 20 s, 63°C for 40 s (with a decreasing temperature step of 0.5°C per cycle), 72°C for 1 min; then followed by 24 cycles of 94°C for 20 s, 65°C for 30 s, 72°C for 1 min; 72°C for 2 min. The primer used for ACAT1 amplification were:

Primer-F: 5’-TAGATTTGGTGGATAYGGGAGTT-3’,

Primer-R: 5’-CAACCCCRCACCCCTAAC-3’

PCR amplicons were diluted and amplified using indexed primers. Specifically, a 20 µl mixture was prepared for each reaction and included 1x reaction buffer (NEB Q5TM), 0.3 mM dNTP, 0.3 µM of F primer, 0.3 µM of index primer, 1 U Q5TM DNA polymerase (NEB), and 1 µL diluted template. The cycling program was 98°C for 30 s; 11 cycles of 98°C for 10 s, 65°C for 30 s, 72°C for 30 s; 72°C for 5 min. PCR amplicons were separated by agarose electrophoresis and purified using QIAquick Gel Extraction kit (QIAGEN). Libraries from different samples were quantified and pooled together, followed by sequencing on the Illumina MiSeq platform according to the manufacturer’s protocols. Sequencing was performed with a 2×150bp paired-end mode.

### Western Blot

Total protein was isolated using RIPA buffer (BeyotimeBiotechnology, China) containing a protease inhibitor cocktail (Fabio Science, China). Equal amounts of protein (50 μg) were separated by electrophoresis on a 4-12% SDS–PAGE gel and transferred to Nitrocellulose Membrane (Thermo Fisher Scientific, USA). The membranes were subsequently blocked in 5% BSA and incubated with primary antibodies overnight at 4°C. Subsequently, secondary antibodies (anti-rabbit/mouse, 1:10000, 926–32211/926–68070, Licor, USA) were used at room temperature for 2 hrs. GAPDH was used as an internal control. Fluorescent signals were captured by LI-COR Odyssey (Li-Cor, USA) imaging system.

### Cell Proliferation Assay

1×10^3^ cells were seeded in each well of 96-well plates. 10 μl CCK-8 reagent (Dojindo, Japan) was added to each well followed by incubation for the indicated periods (1, 2, 3, 4, and 5 days). The absorbance (OD450 nm) was measured in a microplate reader (BioTek, USA). For MTT assay, 10 μl MTT reagent (5mg/ml, Solarbio Science&Technology, China) was added to each well followed by incubation for the indicated periods (1, 2, 3, 4, and 5 days). The absorbance (OD490 nm) was measured in a microplate reader (BioTek, USA).

### *In Vivo* Tumorigenesis

The animal study was approved by the Animal Ethical Committee of First Affiliated Hospital of Guangxi Medical University. All the methods were carried out following the approved guidelines. Five 5-week-old male BALB/c-nu nude mice (Vital River Laboratory Animal Technology, China) were injected with 1×10^6^ 5-8F and HONE1 cells transfected with either pCMV6-Entry or pCMV6-ACAT1 plasmids, in the right flank or the left flank, respectively. The tumor volume was assessed by 2D measurements at 3, 6, 9, 12, 15, 18 days. Tumor volume was calculated as volume (mm^3^) = length×width×0.5. Eighteen days after inoculation for 5-8F cells and fifteen days for HONE1 cells, all mice were killed and tumors were removed.

### cDNA Microarray Analysis

Affymetrix GeneChip Genome U133 Plus 2.0 expression array and Gene-Cloud of Biotechnology Information (GCBI) platform (www.gcbi.com.cn) were used to analyze genes with differential expression between pCMV6-ACAT1-5-8F and pCMV6-Entry-5-8F cells. The microarray data is available *via* the following accession identifier on the NCBI-GEO database: GSE155206.

### Wound Healing Assay

8×10^5^ cells per well were seeded into 12-well plates in DMEM media without FBS overnight. Monolayer cells were scratched by an ibidi Culture-Insert (No. 80209, ibidi, Germany). Images were acquired under an inverted phase microscope (TS100, Nikon, Japan) at 0 h and 24 hrs. The migratory distance was analyzed by Image J ver.1.51k (NIH, USA) software.

### Transwell Assay

7×10^4^ cells per well suspended in 200 μl of serum-free media were seeded in the upper transwell chamber (8 μm pores, Corning, USA), pre-coated with matrigel (BD Biosciences). The lower chamber was filled with DMEM media with 10% FBS. Non-invading cells were removed by using a cotton-tipped swab after 36 hrs. Invasive cells on the lower membrane surface were fixed with 1% paraformaldehyde, stained with 0.5% crystal violet, and photographed.

### β-Hydroxybutyrate Detection

Cells were grown in 60-mm dishes in serum-free DMEM media for 48 hrs and then lysed with RIPA buffer. β-HB colorimetric assay kit (#700190, Cayman Chemical, Ann Arbor, MI, USA) was used for measurement of the intracellular β-HB level as previously (13).

### Statistical Analysis

All data were analyzed using SPSS 20.0 (SPSS Inc., Chicago, IL, USA). The independent Student’s t-test was used to analyze the results and the data expressed as the means ± SD. Statistical significance was considered at *p< 0.05, **p< 0.01 and ***p< 0.001.

## Results

### The Expression of ACAT1 Is Significantly Downregulated in NPC

**Figure 1:**
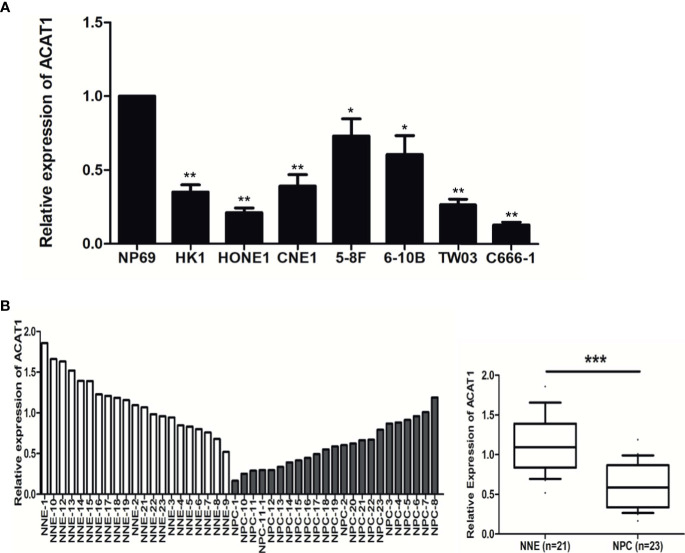
The expression of ACAT1 is downregulated in NPC. **(A)** ACAT1 mRNA level was determined by real-time RT-PCR in 7 NPC cell lines and a non-cancerous nasopharyngeal epithelial (NNE) cell line NP69. The independent Student’s t-test was used to analyze the results and data are expressed as the means ± SD. **(B)** Relative ACAT1 mRNA expression in NPC primary biopsies (n=23) and NNE samples (n=21). The independent Student’s t-test was used to analyze the results. The line inside the boxes represents the median value. The box length indicates the interquartile range. *p < 0.05, **p < 0.01, ***p < 0.001.

**Figure 2:**
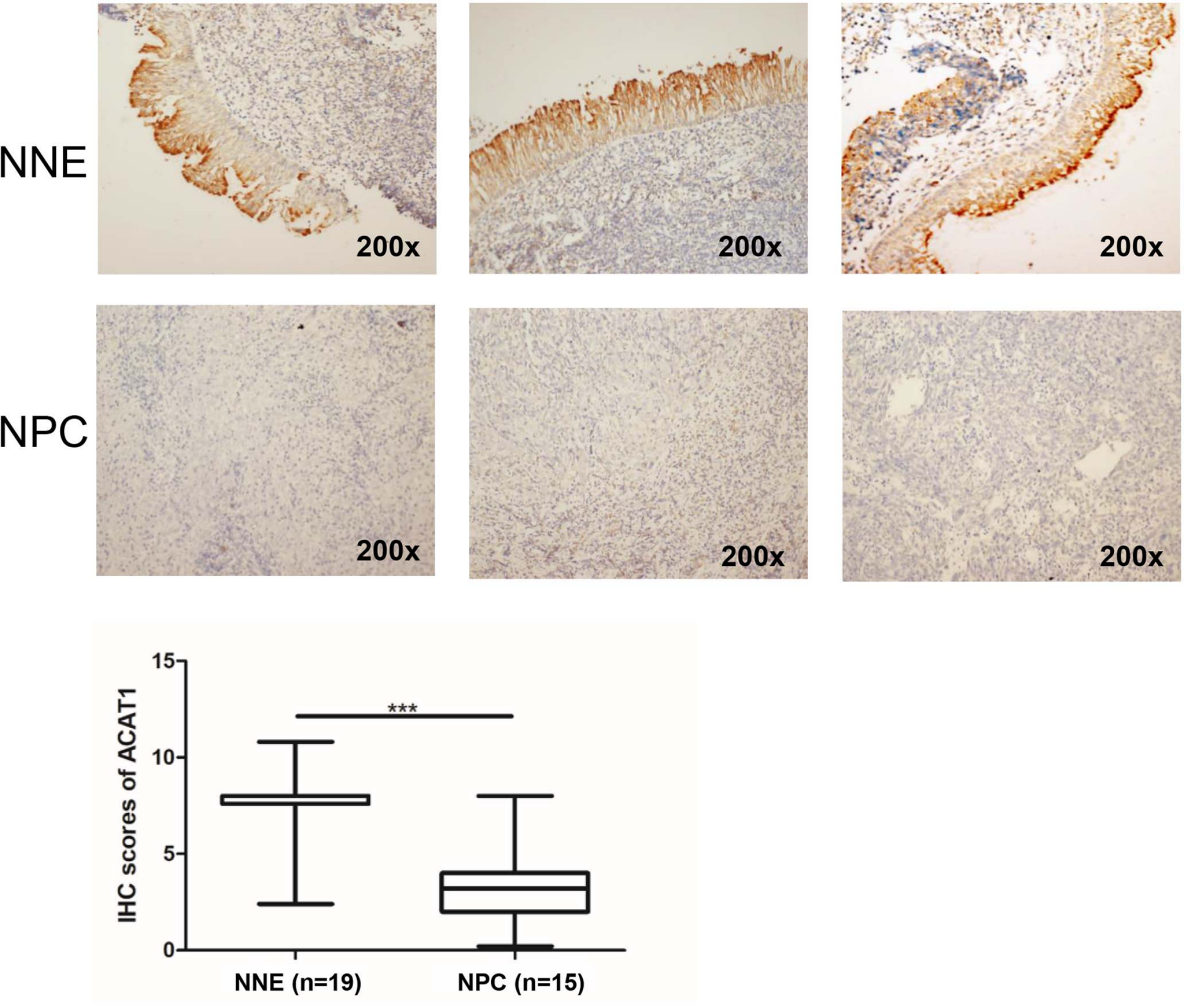
Immunohistochemical staining of ACAT1 in NNE tissue (n=19) and NPC (n=15). Magnifications ×200. The independent Student’s t-test was used to analyze the results. The line inside the boxes represents the median value. The box length indicates the interquartile range. ***p < 0.001.

To reveal the altered expression of ACAT1 in NPC, we firstly investigated the transcription of ACAT1 in seven NPC cell lines (HK1, HONE1, CNE1, 5-8F, 6-10B, and C666-1) compared with an immortalized normal nasopharyngeal epithelial cell line (NP69). **Figure 1A** shows the reduction of ACAT1 in NPC cell lines. Both mRNA and protein expression of ACAT1 in NPC tissues and non-cancer control tissues were also analyzed. The transcription of the ACAT1 was significantly downregulated in 21 NPC primary tumors but was easily detected in all 23 NNE samples (**Figure 1B**). To evaluate the consistency of abnormal transcription of ACAT1 in NPC, we performed a meta-analysis using 6 sets of microarrays from the GEO database including 143 NPC tissues and 42 normal tissues (**Table S1**). The results generated by the random-effects model indicated that significant heterogeneity existed among individual datasets (I^2^ = 61.2%, *p*=0.025) and the pooled Standard Mean Difference (SMD) as -0.98 (95% CI: -1.63,-0.33, **Figure S1A**). No significant difference was found in the sensitivity analysis (**Figure S1B**). The results of Begg’s test showed no significant publication bias (*p*=0.452, **Figure S1C**). Of note, ACAT1 protein was localized in the cytoplasm of cells and was highly expressed in the NNE layer (n=19) (**Figure 2**), while almost absent in NPC tissue (n=15). Thus, these findings support the inactivation of ACAT1in NPC.

### ACAT1 Is Inactivated by DNA Promoter Hypermethylation in NPC

**Figure 3:**
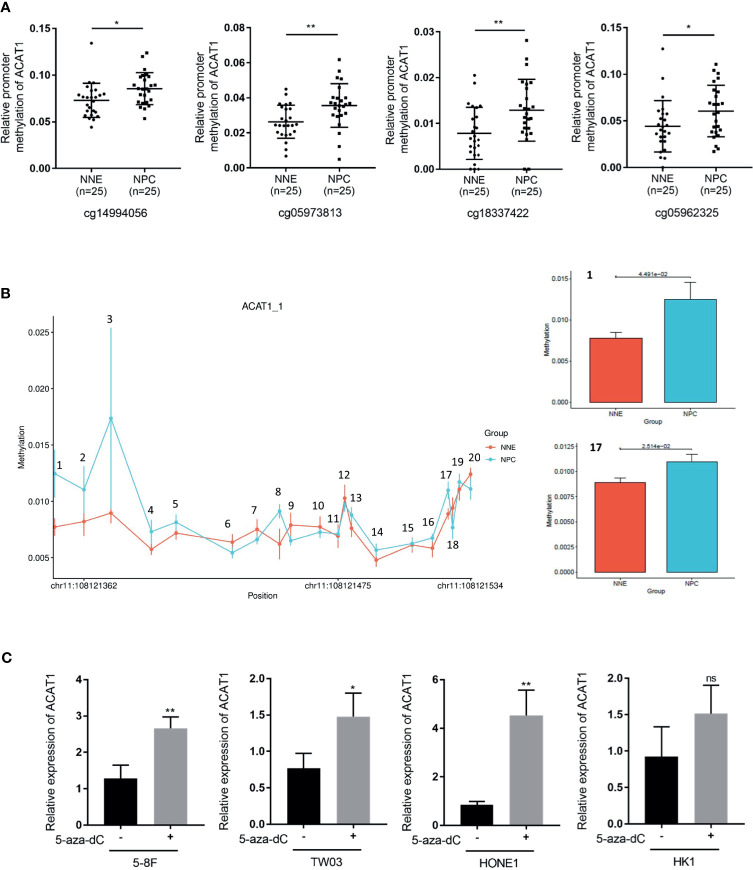
DNA hypermethylation of the ACAT1 CpG island in NPC tumor biopsies and normal nasopharyngeal tissue. **(A)** DNA methylation microarray data (GSE62336) containing 25 cases of NPC primary tissues and 25 cases of normal nasopharyngeal epitheliums was used for analyzing the relative methylation level in the CpG island of the ACAT1 gene. Four probes targeting the CpG island region of ACAT1 were found. The dot plot shows the relative methylation level of the ACAT1 gene. **(B)** The methylation status of the 20 CpG sites within the ACAT1 gene promoter in 20 NPC biopsies and 9 normal nasopharyngeal epithelium biopsies were analyzed by bisulfite genomic sequencing. **(C)** Real-time RT-PCR analysis of the mRNA level of ACAT1 in four NPC cell lines with and without treatment with 5-aza-dC, 5μM/L for 4 days. The independent Student’s t-test was used to analyze the results and data are expressed as the means ± SD. *p < 0.05, **p < 0.01.

Interestingly, a CpG island with a length of 241bp (-295bp~-55bp from the transcription starting site) was found in the DNA promoter region of ACAT1. We analyzed the methylation microarray dataset (GSE62336) and found that the methylation modification in this CpG island region of ACAT1 is remarkably stronger in NPC (n=25) in contrast to NNE (n=25) tissues (**Figure 3A**). To confirm this, a bisulfite sequencing of the ACAT1 promoter region (-217bp~-32bp) was carried out in NPC primary tissues (n=20) and NNE control samples (n=9). This region includes 20 CpG sites, covering the probe of cg05973813 and cg18337422 shown in **Figure 3A**. We found that the average methylation ratio was higher in 11 out of 20 CpG sites in NPC samples, in contrast with normal samples (**Figure 3B**). Among them, two CpG sites were significantly more methylated in NPC. In addition, the mRNA level of ACAT1 was restored significantly upon treatment with the demethylation reagent 5-aza-dC in the NPC cell lines 5-8F, HONE1, and TW03. The mRNA level of ACAT1 was elevated in HK1 after 5-aza-dC treatment as well, but no significant difference was observed (**Figure 3C**). These data suggest that ACAT1 might be inactivated in NPC by DNA promoter hypermethylation.

### Overexpression of ACAT1 Suppresses NPC Cell Proliferation and Colony Formation *In Vitro*

**Figure 4:**
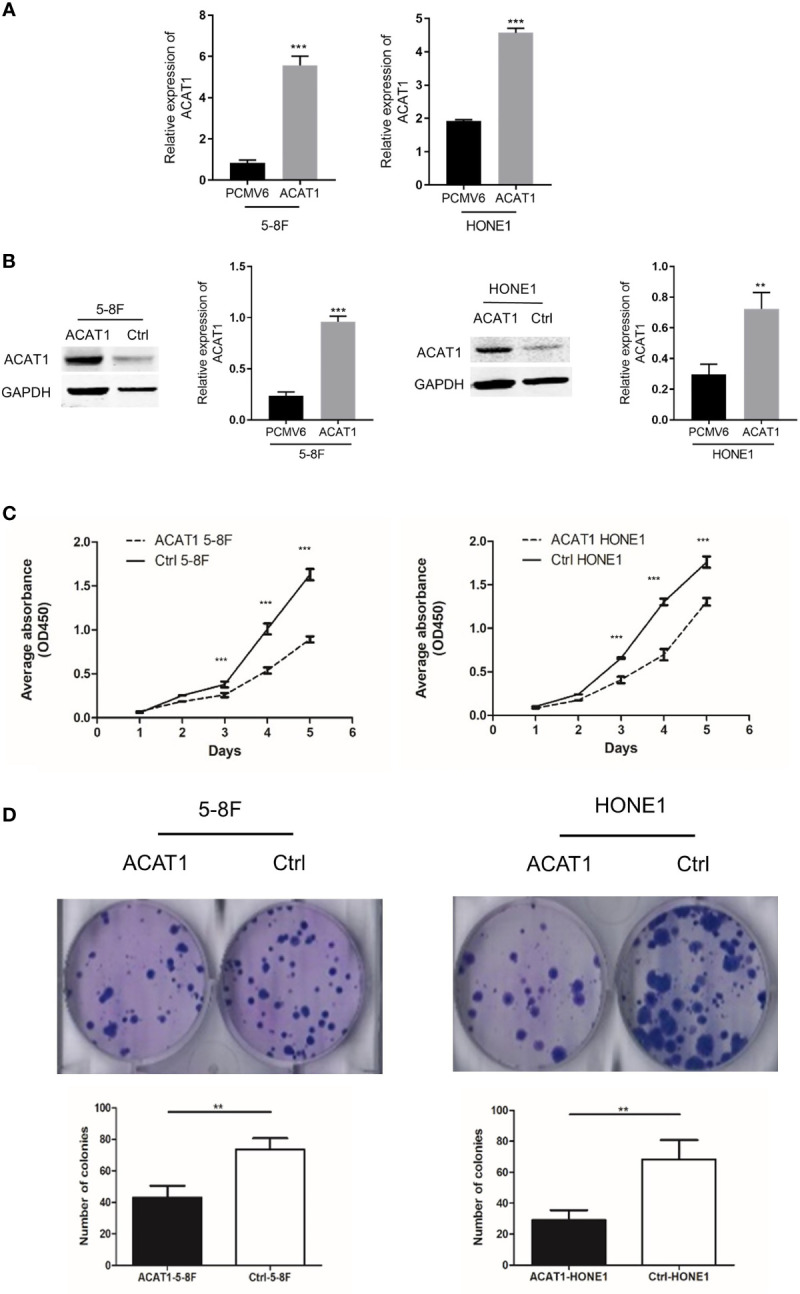
Overexpression of ACAT1 inhibits proliferation. **(A, B)** ACAT1 expression in stably transfected 5-8F and HONE1 cell lines was confirmed by qPCR and western blotting, respectively. **(C, D)** Proliferation and colony formation of ACAT1-5-8F and ACAT1-HONE1 cells determined by CCK-8 assay (OD=450nm) and colony formation assay, respectively. The independent Student’s t-test was used to analyze the results and data are expressed as the means ± SD. **p < 0.01; ***p < 0.001.

We established 5-8F and HONE1 cell lines stably overexpressing ACAT1 to investigate the biological function of ACAT1 in NPC (**Figure 4A**). Western blot results confirmed that ACAT1 was successfully expressed in both cell lines (**Figure 4B**). The proliferative rate of ACAT1-5-8F and ACAT1-HONE1 cells was remarkably slower than the pCMV6-Entry control cells (**Figure 4C**). Also, the capacity of colony formation of both NPC cell lines was reduced significantly by ACAT1 overexpression (**Figure 4D**). Therefore, these indicate that overexpressing ACAT1 suppresses the growth of NPC cells *in vitro*.

### ACAT1 Suppresses NPC Tumor Formation *In Vivo*

**Figure 5:**
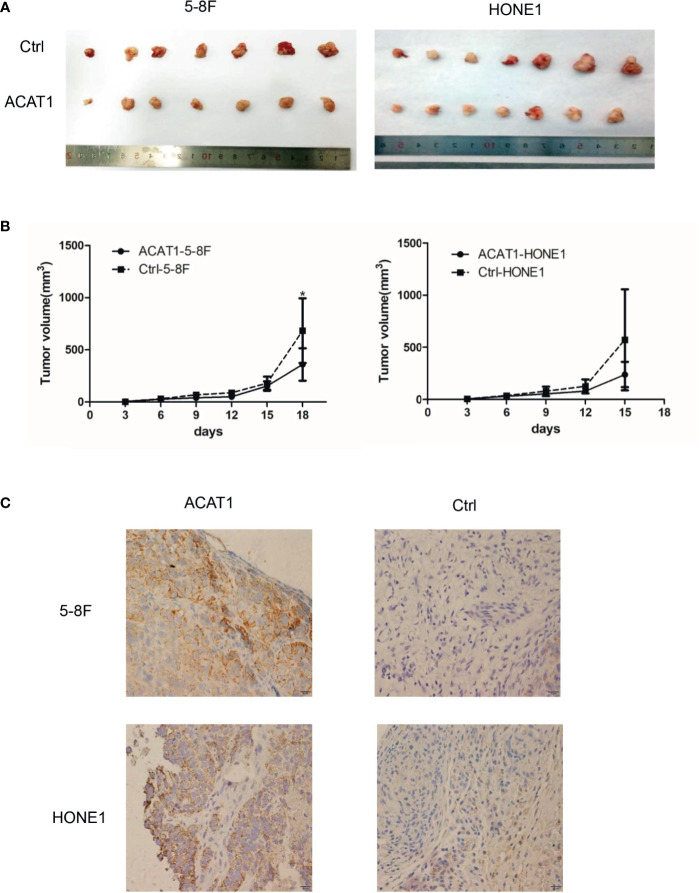
ACAT1 suppresses tumorigenesis of NPC cells in *vivo*. **(A)** Xenografts in nude mice from inoculated ACAT1-5-8F/HONE1 and pCMV6-Entry-5-8F cells were removed at day 18 (5-8F) and day 15 (HONE1) **(B)** Volume of the tumors measured at 0, 3, 6, 9, 12, 15, 18 days after inoculation. **(C)** Immunohistochemistry staining was used to determine the expression of ACAT1 in xenografts. Magnifications ×400. The independent Student’s t-test was used to analyze the results and data are expressed as the means ± SD. *p < 0.05.

To further evaluate the suppressive effect of ACAT1 expression on NPC tumor cell growth, we compared the tumorigenesis of ACAT1-5-8F and ACAT1-HONE1 to that of the respective pCMV6-Entry control clones of 5-8F and HONE1 *in vivo*. The average volume of tumors derived from ACAT1-5-8F and ACAT1-HONE1 is lower than that from pCMV6-Entry clones (**Figure 5**). However, a statistical significance was observed only in the 5-8F cell line.

### ACAT1 Suppresses NPC Cell Migration and Invasion *via* Epithelial-Mesenchymal Transition

**Figure 6:**
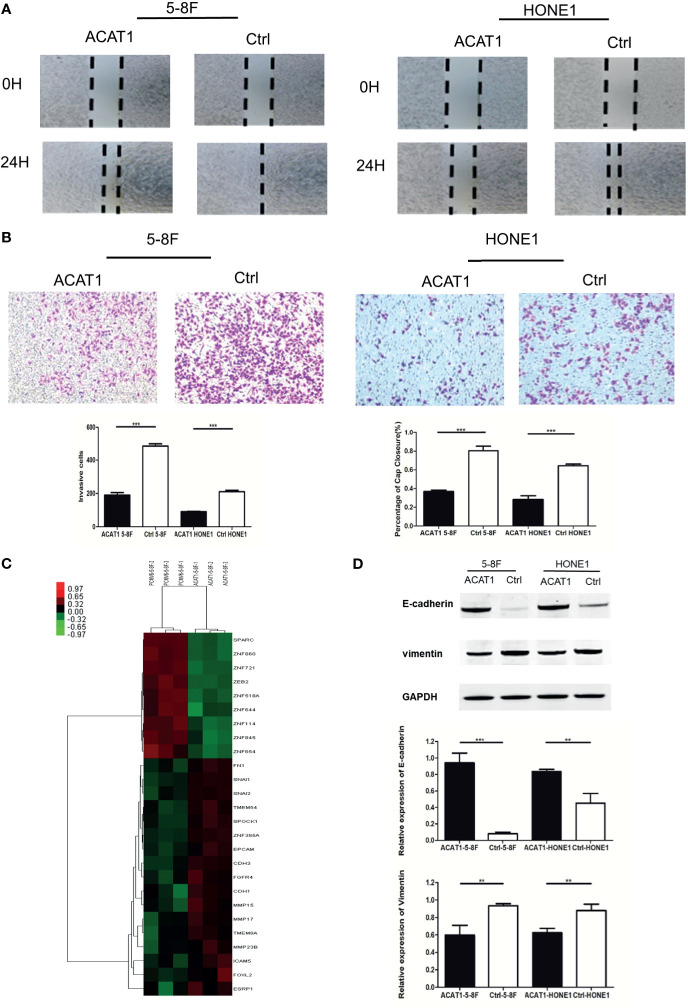
Overexpression of ACAT1 suppresses the migration and invasion of NPC cell lines *in vitro* by reversing EMT. **(A)** Migration of NPC cells stably transfected with ACAT1 or empty vector was examined by a wound-healing assay. The gap closure was photographed and measured at 0 and 24 h. The percentage of wound width for each sample was calculated by ImageJ software. Magnification ×100. **(B)** Invasion of NPC cells stably transfected with ACAT1 or empty vector was examined by transwell assay. The blue dots represent the invading cells stained with crystal violet. The number of invading cells was counted and is shown in the bar graph. **(C)** cDNA microarray data: heatmap showing expression of 26 genes involved in EMT in ACAT1-overexpressing 5-8F cells as compared with pCMV6-Entry-5-8F cells. **(D)** The expression of E-cadherin and Vimentin was detected by western blot and quantitated by densitometric scanning. GAPDH was used as an internal control. Data are expressed as means ± s.d. in a bar graph. The independent Student’s t-test was used to analyze the results and data are expressed as the means ± SD. **p < 0.01, ***p < 0.001.

We further addressed the effect of ACAT1 on migratory and invasive capacities in NPC cells. The wound healing assays revealed a slower gap closure in ACAT1-5-8F and ACAT1-HONE1 cells in contrast to control cell lines (**Figure 6A**). Besides, the number of invading cells in the ACAT1 expressing clones was lower than for the control cell lines (**Figure 6B**). Our finding suggests that overexpression of ACAT1 attenuates the migration and invasion of NPC cells.

To investigate the potential molecular mechanism by which ACAT1 affects migratory and invasive capacity, we performed cDNA microarray analysis in ACAT1-5-8F and PCMV6-Entry-5-8F cells. We found that genes involved in epithelial-mesenchymal transition (EMT) were significantly altered by ACAT1 overexpression, including downregulation of SPARC, ZEB2, etc. and upregulation of EPCAM, CDH1, and CDH3, etc. (**Figure 6C** and **Figure S2**). In addition, we analyzed the expression of E-cadherin and vimentin by western blot. In comparison with control cell lines, E-cadherin was upregulated in ACAT1-5-8F and ACAT1-HONE1 cells, while vimentin was downregulated (**Figure 6D**). Therefore, overexpression of ACAT1 may reverse the EMT process, thereby reducing the metastatic potential of NPC cells.

### ACAT1 Increases the Intracellular Level of β-Hydroxybutyrate in NPC Cells

**Figure 7:**
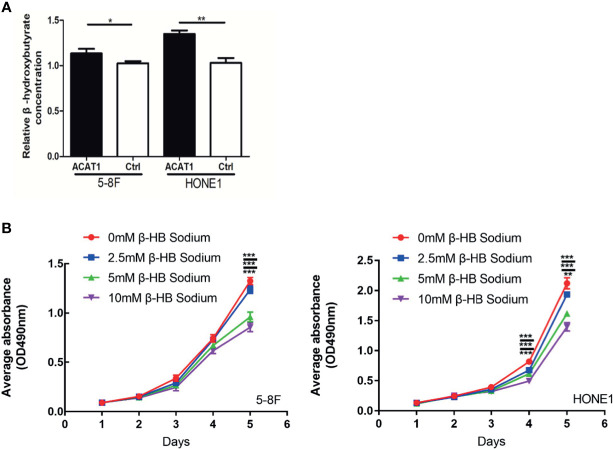
ACAT1 elevates the intracellular level of β-hydroxybutyrate (β-HB) in NPC cells. **(A)** The relative concentration of intracellular β-HB in ACAT1-5-8F/HONE1 and Ctrl-5-8F/HONE1 cells. **(B)** MTT assay was performed to measure the proliferation of 5-8F and HONE1 cells after β-HB treatment at 0 mM, 2.5 mM, 5 mM, and 10 mM. The independent Student’s t-test was used to analyze the results and data are expressed as the means ± SD (n=5). *p < 0.05; **p < 0.01; ***p < 0.001.

As a key enzyme for ketogenesis, the regulation of β-HB by ACAT1 was investigated. We assessed the relative concentration of intracellular β-HB, the main component of ketone bodies, in ACAT1-expressing 5-8F and HONE1 cells compared to pCMV6-Entry-control cells. The intracellular β-HB level was observed significantly higher in ACAT1-expressing cells than in pCMV6-Entry control cells (**Figure 7A**). To further address if ACAT1 inhibits the proliferation of NPC cells *via* elevating β-HB production, we treated NPC cells with exogenous β-HB. The extracellular β-HB suppressed the proliferation of HONE1 and 5-8F cells in a dose-dependent manner (**Figure 7B**), which was in line with our previous finding in other NPC cell lines (13). Thus, overexpression of ACAT1 might reverse the malignant phenotype of NPC cells by increasing β-HB.

## Discussion

Functioning as high-energy mitochondrial fuels, ketone bodies are normally generated in hepatocytes and used during starvation (19). Recent studies provide evidence that ketone bodies can be produced in cancer cells as well as in tumor stroma (13, 20). In this study, we show that the enzyme ACAT1, catalyzing the first step of ketogenesis, is inactivated in NPC cell lines and primary tumor tissues. This further supports our previous finding that the production of ketones in NPC is inhibited. However, ACAT1 is also required for the utilization of ketone bodies. This suggests that NPC cells use other energy sources than ketones to provide the energy needed for promoting growth, such as glycolysis (21), uptaking glutamine (22), fatty acid oxidation (23), and so on,. Thus, a pattern of inactivated ketone body metabolism remains one of the significant hallmarks of NPC. In addition to ACAT1, the other two key enzymes in ketone body metabolism, BDH1 and OXT1, are also downregulated in glioblastoma (24). It is known that the expression of ACAT1 is regulated by the well-studied onco-microRNA miR-21, which is elevated in various tumors (25). We found a CpG island in the promoter region of ACAT1. Analysis of the methylation microarray data from the GEO database suggests that the methylation level of this CpG island region is significantly higher in NPC tissues in contrast to normal control tissues. Our bisulfite sequencing data confirmed significant hypermethylation of 2 CpG sites in this CpG island in NPC tissues. In addition, the 5-aza-dC treatment resulted in upregulation of ACAT1 at the mRNA level, further indicating that ACAT1 is inactivated by DNA hypermethylation in NPC. To our knowledge, this is a novel mechanism for modulating ACAT1 expression in tumors. The upregulation of ACAT1 in breast cancer was shown to induce apoptosis (25). We found that restoring the expression of ACAT1 in NPC leads to increased intracellular β-HB levels with a concomitant decrease in proliferation, colony formation, and *in vivo* tumorigenesis. The growth of NPC cells was also impeded by the presence of extracellular β-HB. This directly supports the notion that ketones interfere with the survival of NPC cells. Previously, it was shown that both intracellular and extracellular β-HB levels induce the significant generation of ROS, thereby suppressing the viability of NPC (13). β-HB may exert it *via* inhibition of NLRP3 inflammasome complex assembly (26, 27). However, ROS species are also generated during inflammation, and chronic inflammation is recognized as a contributing factor for tumorigenesis. It promotes tumor development, progression, and metastatic dissemination, as well as treatment resistance. Thus, in glioma, treatment with β-HB inhibits the inflammatory microenvironment, resulting in suppression of tumor cell migration (28). In line with this, our study shows that an increase in β-HB levels due to restoration of ACAT1 expression results in decreased motility of NPC cells.

The role of ketone body metabolism in tumorigenesis has received prior attention. 3-Hydroxymethylglutaryl-CoA synthase 2 (HMGCS2) is the enzyme directly downstream of ACAT1 in the ketogenesis pathway (29). Its inactivation in hepatocellular carcinoma has been verified and was positively associated with ketone body production. Importantly, the ketone bodies inhibited hepatocellular carcinoma cell migration by reversing epithelial-mesenchymal transition (EMT) signaling (30). In line with this, we showed that the epithelial markers (CDH1 and EPCAM) were increased in NPC cells, in which expression of ACAT1 was restored, while mesenchymal markers (vimentin and SPARC) were decreased. Our data indicate that ACAT1 represses the movement of NPC cells by regulating the expression of EMT-related markers.

To further uncover the mechanisms underlying the ACAT1-mediated control of the EMT signaling in NPC cells, we focused on CDH1, the expression of which is upregulated by either overexpression of ACAT1 or by β-HB treatment (13). Acting as an endogenous histone deacetylase inhibitor (HDACi), β-HB can have a systemic effect, regulating the expression of a multitude of genes, affecting several cellular processes (31). We observed increased acetylation of lysine 9 and lysine 14 of histone 3 in the promoter region of CDH1 after the treatment with β-HB, indicating transcriptional stimulation of CDH1 (data not shown). Previously, HDACi induced p53-dependent apoptosis in NPC cells (32) and retarded the growth of carcinomas of the cervix, colon, and rectum *in vitro* (33). Surprisingly, a recent study reported that NPC cells were turned into a mesenchymal cell phenotype after short-term stimulation with the HDACi trichostatin A. However, an invasive phenotype was not induced (34). Additionally, HDACs contribute to maintaining EBV latent infection (35), whereas HDACis, such as Trichostatin A (TSA), Suberoylanilide Hydroxamic acid (SAHA), and butyric acid, act as potent inducers of EBV reactivation, as well as mediating apoptosis of NPC cells. Therefore, reactivating EBV by HDACi is a potential approach to treat NPC. So far, it remains unknown if EBV reactivation in NPC cell lines can be induced by β-HB. Further verification is necessary.

In fact, as non-toxic adjuvant therapy, ketogenic diets or ketone supplementation have shown a positive therapeutic advantage in malignancies. Notably, the preclinical studies demonstrate that a ketogenic diet increases the radiation sensitivity in xenograft models of pancreatic cancer (36). Our previous and present findings show that overexpressing the ketogenesis genes HMGCL and ACAT1, resulted in increased intracellular β-HB in both renal cell carcinoma and NPC (13, 16), thereby inhibiting the growth capacity of tumor cells. As well, exogenous β-HB remarkably suppresses the proliferation and metastasis of NPC cells. Thus, elevating the level of β-HB by targeting ketogenic genes, ketogenic diets or ketone supplementation might be a promising therapeutic approach for NPC patients. This is worthy of further confirmation in animal models.

In summary, we discovered that the expression of ACAT1 was inactivated in NPC due to promoter hypermethylation. Overexpression of ACAT1 elevated the intracellular β-HB levels in NPC cells, inhibited the proliferation, migration, and invasive growth of NPC cells. Our data reveal that epigenetic modification affects the ketone body metabolism and that this dysregulation might contribute to the pathogenesis of NPC. In addition, our findings support a therapeutic approach to manipulate ketone body metabolism, for example, through the addition of drugs or dietary intervention, as a potentially useful approach for the prevention and treatment of NPC.

## Data Availability Statement

The datasets presented in this study can be found in online repositories. The names of the repository/repositories and accession number(s) can be found in the article/**Supplementary Material**.

## Ethics Statement

The study was reviewed and approved by Research Ethics Committee of the First Affiliated Hospital of Guangxi Medical University, China (documents no.2016-KY-050).

## Author Contributions

YL, XiaohZ, and PH performed the experiments. BL, ZL, YY and XuemZ analyzed the data. XX, YM, and PL provided research materials and methods. YL, XiaohZ, and WZ wrote the manuscript. XiaoyZ, ZZ, and GH conceived the study and revised the manuscript. All authors contributed to the article and approved the submitted version.

## Funding

This work was supported by grants from the National Natural Science Foundation of China (81460412, 81772882, 81660458, 81660445).

## Conflict of Interest

The authors declare that the research was conducted in the absence of any commercial or financial relationships that could be construed as a potential conflict of interest.

## Publisher’s Note

All claims expressed in this article are solely those of the authors and do not necessarily represent those of their affiliated organizations, or those of the publisher, the editors and the reviewers. Any product that may be evaluated in this article, or claim that may be made by its manufacturer, is not guaranteed or endorsed by the publisher.

## Abbreviations

5-aza-dC, 5-aza-2’-deoxycytidine; ACAT1, Acetyl-CoA acetyltransferase 1; BDH2, 3-hydroxybutyrate dehydrogenase type 2; β-HB, β-hydroxybutyrate; EBV, Epstein-Barr Virus; EMT, epithelial-mesenchymal transition; HDACi, histone deacetylases inhibitor; HMGCL, hydroxymethylglutaryl-CoA lyase; NPC, nasopharyngeal carcinoma.
