# Supplementary material for: Epigenetic Inactivation of Acetyl-CoA Acetyltransferase 1 Promotes the Proliferation and Metastasis in Nasopharyngeal Carcinoma by Blocking Ketogenesis
Source: Front Oncol. 2021 Aug 16;11:667673. doi: 10.3389/fonc.2021.667673 (PMC8415477; doi:10.3389/fonc.2021.667673)

**Supplementary Material and Methods**

**A meta-analysis based on the GEO database**

The cDNA microarray data comparing the transcriptional level of ACAT1 in NPC and normal nasopharyngeal epithelium were obtained from the GEO database (<http://www.ncbi.nlm.nih.gov/geo>), with search terms as follow: (nasopharyngeal OR nasopharynx) AND (cancer OR carcinoma OR adenocarcinoma OR tumour OR tumor OR malignancy/malignant* OR neoplasm* OR oncology*). The criteria of inclusion were: (1) gene expression data were extracted from homo sapiens; (2) samples were all obtained from malignant tissues or non-cancerous NPC tissues; (3) both healthy and NPC groups were comprised of at least three cases; (4) patients involved did not receive treatment. The STATA 12 software was used for meta-analysis.

**Figure S1**


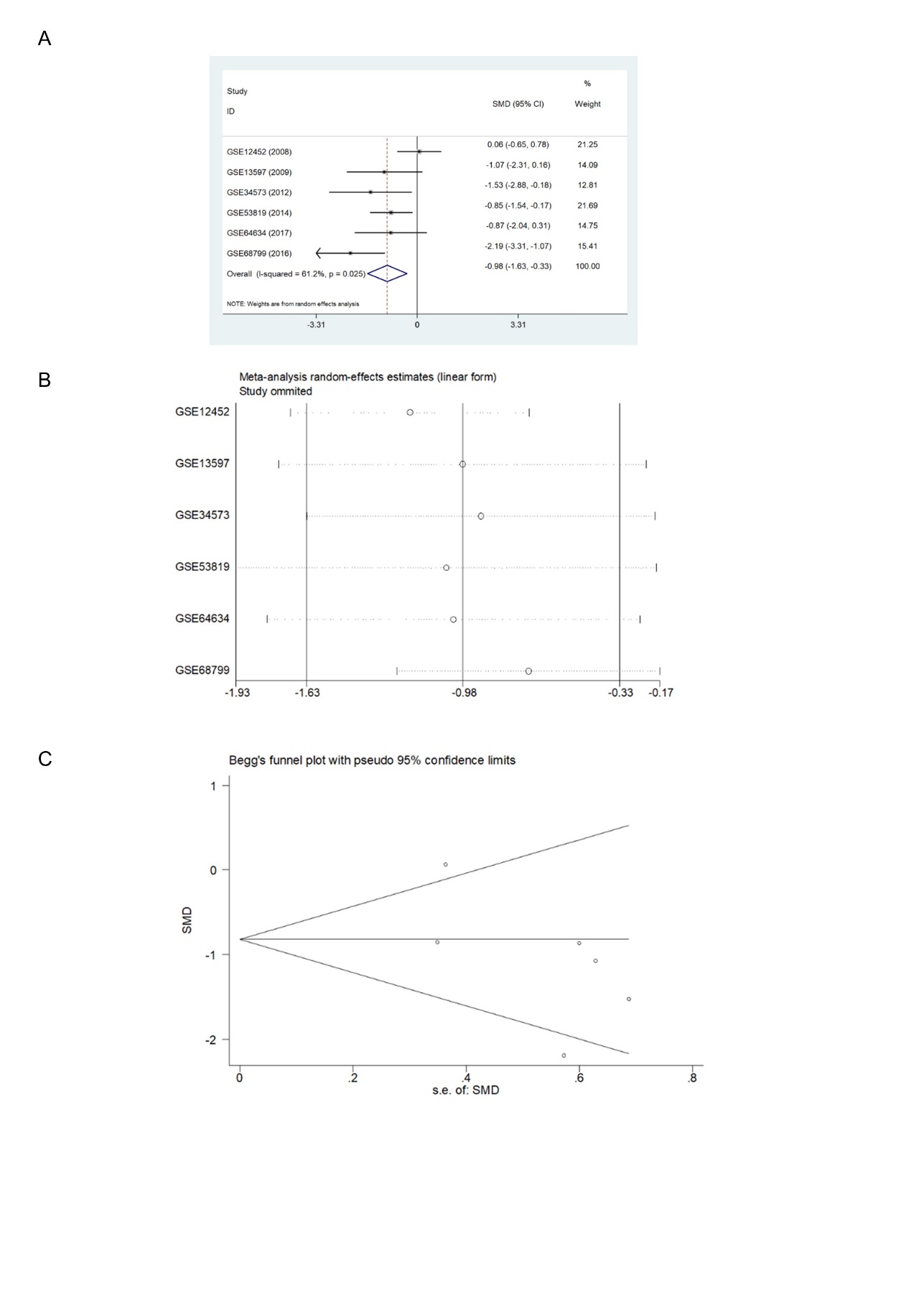


**Figure legend S1:** **Meta-analysis revealing the downregulation of ACAT1 in NPC patients compared with normal cases.** (A) Forest plot of ACAT1 expression in NPC and normal control cases. (B) Sensitivity analysis of meta-analysis for ACAT1. (C) Begg’s publication test plot of meta-analysis for ACAT1.

**Figure S2**

**
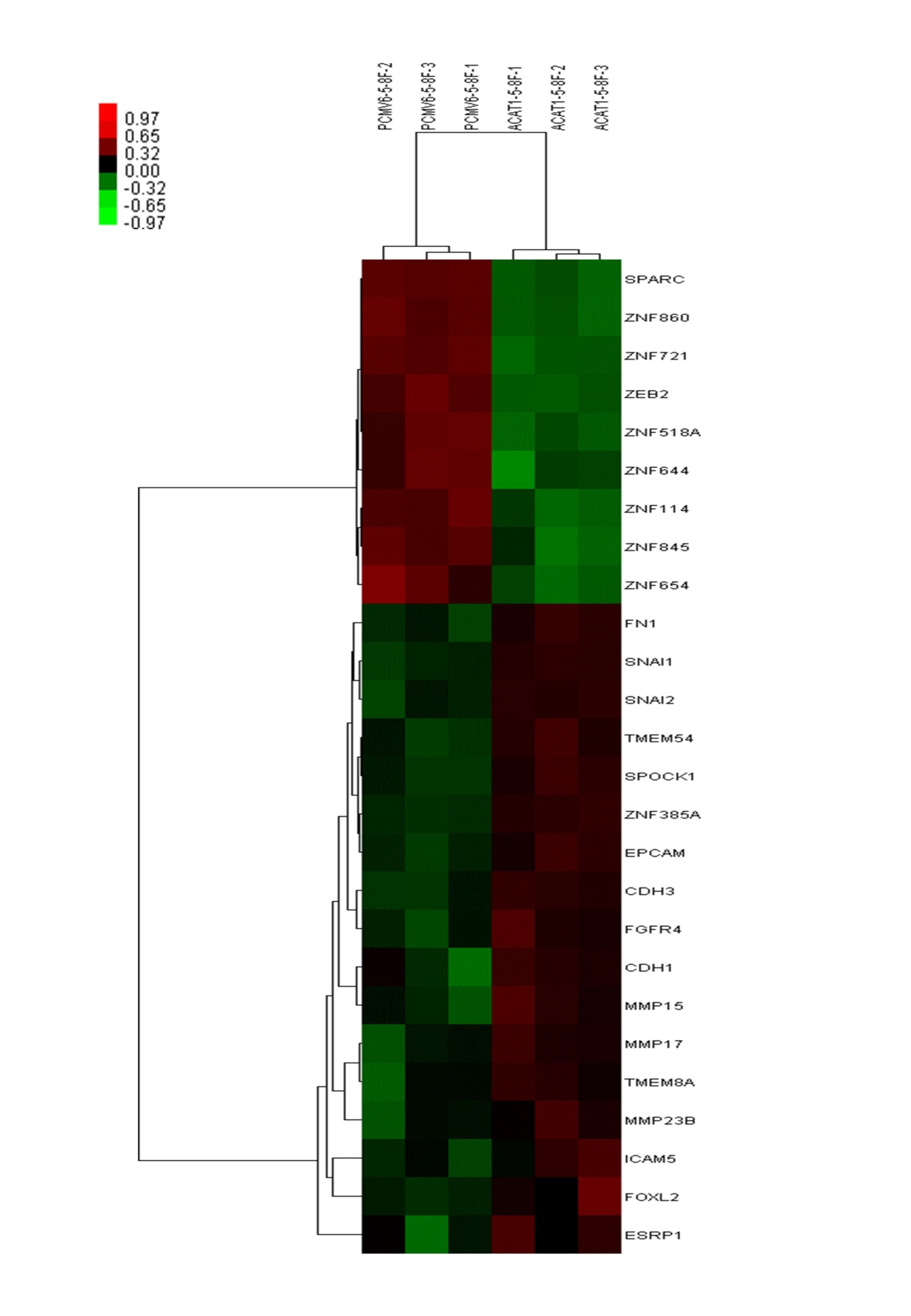
**

**Figure legend S2:** cDNA microarray data: heatmap showing expression of 26 genes involved in EMT in ACAT1-overexpressing 5-8F cells as compared with pCMV6-Entry-5-8F cells.

**Table S1**

**Table S1. Detailed information on the GEO database used for Meta-analysis.**


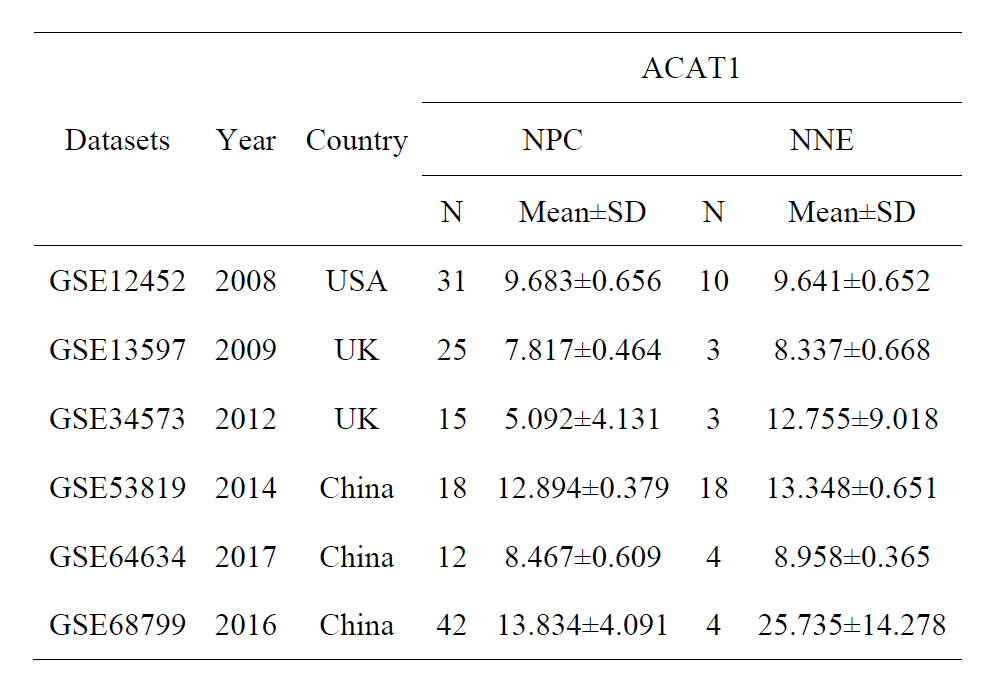

Supplement: Supplementary file 1 [file DataSheet_1.docx]
